# The binding characteristics and orientation of a novel radioligand with distinct properties at 5-HT_3_A and 5-HT_3_AB receptors

**DOI:** 10.1016/j.neuropharm.2014.08.008

**Published:** 2014-11

**Authors:** Andrew J. Thompson, Mark H.P. Verheij, Joost Verbeek, Albert D. Windhorst, Iwan J.P. de Esch, Sarah C.R. Lummis

**Affiliations:** a Amsterdam Institute for Molecules Medicines and Systems (AIMMS), Division of Medicinal Chemistry, Faculty of Sciences, VU University Amsterdam, Amsterdam, The Netherlands; b Department of Biochemistry, University of Cambridge, Cambridge, UK; c VU University Medical Center, Dept Radiology & Nuclear Medicine, Amsterdam, The Netherlands

**Keywords:** Antagonist, Cys-loop, Ion channel, Radioligand, 5-HT_3_, Agonist, 5-HT, 5-hydroxytryptamine, nACh, nicotinic acetylcholine, GABA, gamma-aminobutyric acid, HEK, human embryonic kidney, AChBP, acetylcholine binding protein, 5HTBP, an AChBP mutant modified to resemble the 5-HT_3_R binding site, VUF101066, 2-chloro-3-(4-methylpiperazin-1-yl)quinoxaline

## Abstract

VUF10166 (2-chloro-3-(4-methyl piperazin-1-yl)quinoxaline) is a ligand that binds with high affinity to 5-HT_3_ receptors. Here we synthesise [^3^H]VUF10166 and characterise its binding properties at 5-HT_3_A and 5-HT_3_AB receptors. At 5-HT_3_A receptors [^3^H]VUF10166 displayed saturable binding with a *K*_d_ of 0.18 nM. Kinetic measurements gave monophasic association (6.25 × 10^7^ M^−1^ min^−1^) and dissociation (0.01 min^−1^) rates that yielded a similar *K*_d_ value (0.16 nM). At 5-HT_3_AB receptors two association (6.15 × 10^−7^, 7.23 M^−1^ min^−1^) and dissociation (0.024, 0.162 min^−1^) rates were seen, yielding *K*_d_ values (0.38 nM and 22 nM) that were consistent with values obtained in saturation (*K*_d_ = 0.74 nM) and competition (*K*_i_ = 37 nM) binding experiments respectively. At both receptor types, specific binding was inhibited by classical 5-HT_3_ receptor-selective orthosteric ligands (5-HT, allosetron, *d*-tubocurarine, granisetron, *m*CPBG, MDL72222, quipazine), but not by non-competitive antagonists (bilobalide, ginkgolide B, picrotoxin) or competitive ligands of other Cys-loop receptors (ACh, bicuculline, glycine, gabazine). To explore VUF10166 ligand–receptor interactions we used *in silico* modelling and docking, and tested the predictions using site directed mutagenesis. The data suggest that VUF10166 adopts a similar orientation to 5-HT_3_ receptor agonists bound in AChBP (varenicline) and 5HTBP (5-HT) crystal structures.

## Highlights

• [^3^H]VUF10166 binds specifically to 5-HT_3_A and 5-HT_3_AB receptors.
• Association and dissociation rates are biphasic only at 5-HT_3_AB receptors.
• Only competitive 5-HT_3_ receptor-selective compounds inhibit [^3^H]VUF10166 binding.
• VUF10166 likely binds to both an A+A− and an A+B− interface.
• Mutations and docking indicate an agonist-like VUF10166 binding site orientation.

## 1 Introduction

5-HT_3_ receptors are transmembrane ligand-gated ion-channels that are responsible for fast synaptic neurotransmission in the central and peripheral nervous systems. They are composed of five subunits, each of which contains an extracellular, a transmembrane and an intracellular domain (Thompson et al., 2008a; Miller and Smart, 2012). *In vivo* 5-HT_3_ receptor activation can result in nausea and vomiting, and for over three decades competitive antagonists of these receptors have been used to alleviate these symptoms arising from cancer therapy and general anaesthetics. There is also a limited use of antagonists for treating irritable bowel syndrome and pre-clinical interest in the use of partial agonists for the same disorder (Thompson and Lummis, 2007, Walstab et al., 2010, Thompson, 2013).

There are currently five 5-HT_3_ receptor subunits (5-HT3A–5-HT3E), with further complexity arising from splice variants and species differences (Walstab et al., 2010). 5-HT3A subunits can form homomeric receptors, but the subunits 5-HT3B–5-HT3E must combine with 5-HT3A subunits to function. The functional properties of these receptor subtypes have been reported by several groups, but to date only the pharmacologies of 5-HT_3_A and 5-HT_3_AB receptors have been studied in detail (Holbrook et al., 2009, Walstab et al., 2010, Thompson et al., 2013, Thompson and Lummis, 2013). Until recently only pore-blocking antagonists were known to have different properties at 5-HT_3_A and 5-HT_3_AB receptors, and these differences could be attributed to the varying pore-lining amino acids of the 5-HT3A and 5-HT3B subunits (Thompson and Lummis, 2013). However, the utility of these compounds is limited as they tend to be of low affinity (μM range) and also target other receptor types. More recently there have been descriptions of two compounds with other sites of action that discriminate between 5-HT_3_A and 5-HT_3_AB receptor subtypes. One of these, topotecan, primarily an anticancer drug, was found to inhibit 5-HT_3_A and potentiate 5-HT_3_AB receptors, although this compound also has a relatively low (μM) potency (Nakamura et al., 2013). The second compound is VUF10166 (2-chloro-3-(4-methylpiperazin-1-yl)quinoxaline), which is highly potent, with an affinity at 5-HT_3_A receptors (p*K*_i_ ∼ 10) that is ∼100-fold greater than at 5-HT_3_AB receptors (Thompson et al., 2012). We previously showed that VUF10166 binds to the orthosteric binding site of both 5-HT_3_A and 5-HT_3_AB receptors (formed at the interface of two 5-HT3A subunits, A+A−) and that a second, allosteric, binding site (A+B−) in the 5-HT_3_AB receptor was responsible for causing ligands at the A+A− binding site to dissociate more rapidly.

Here we perform a detailed characterisation of VUF10166 binding to 5-HT_3_A and 5-HT_3_AB receptors with a radiolabelled version of this compound and use mutagenesis to explore the residues that interact with VUF10166 at the A+A− binding site.

## 2 Experimental procedures

### 2.1 Synthesis of [^3^H]VUF10166

60 μl [^3^H]methyl nosylate (0.7 GBq/ml, 19 mCi/ml) in hexane/ethyl acetate (10/2 v/v) was injected into a closed reaction screwcap reaction vessel and the solvent evaporated under argon at 60 °C. 2-chloro-3-(piperazin-1-yl)quinoxaline hydrochloride (7.2 mg, 0.025 mmol) in dry DMF (150 μl) and DIPEA (30.7 μL, 0.176 mmol) were added for 1 h at room temperature. The reaction was quenched with 500 μl semi-prep HPLC eluent and subjected to semi-preparative HPLC purification, using a Reprosphere C18-DE 5 μM, 50*8 mm column as stationary phase (Dr. Maisch, Ammerbuch-Entringen, Germany) and acetonitrile/water 75/25 (v/v) with 0.1% diisopropylethylamine as eluent at a flow of 3 ml min^−1^, with UV monitoring at 254 nm (Jasco UV-1575, Jasco, de Meern, Netherlands). 30 s fractions were collected, 5 μl of each added to 5 ml scintillation fluid, and counted for 1 min in a beta well counter (Rackbeta 1219 LSC, LKB-Wallac, Netherlands). Fractions containing 2-chloro-3-(4-[^3^H]methylpiperazin-1-yl)quinoxaline were diluted with 45 ml sterile water and passed over a preconditioned Waters tC18 plus Sep-Pak, washed with 20 ml of water, and the product obtained by elution with 1.5 ml ethanol; 35 MBq (83% radiochemical yield) of [^3^H]VUF10166 was obtained. The specific activity of the product was 3.13 TBq/mmol (84.5 Ci/mmol) and the radiochemical purity was >98%, as determined by HPLC with a Platinum C18 100a, 5 μM 250*4.6 mm column (Grace Alltech, Breda, Netherlands) as stationary phase and acetonitrile/10 mM ammoniumdihydrogen phosphate buffer pH 2.5 50/50 (v/v) as eluent at a flow of 1 ml min^−1^, with UV monitoring at 254 nm (Jasco UV-1575) and radioactivity monitoring (Lablogic β-RAM model 4, Metorix, Goedereede, Netherlands).

### 2.2 Site-directed mutagenesis

**Fig. 1:**
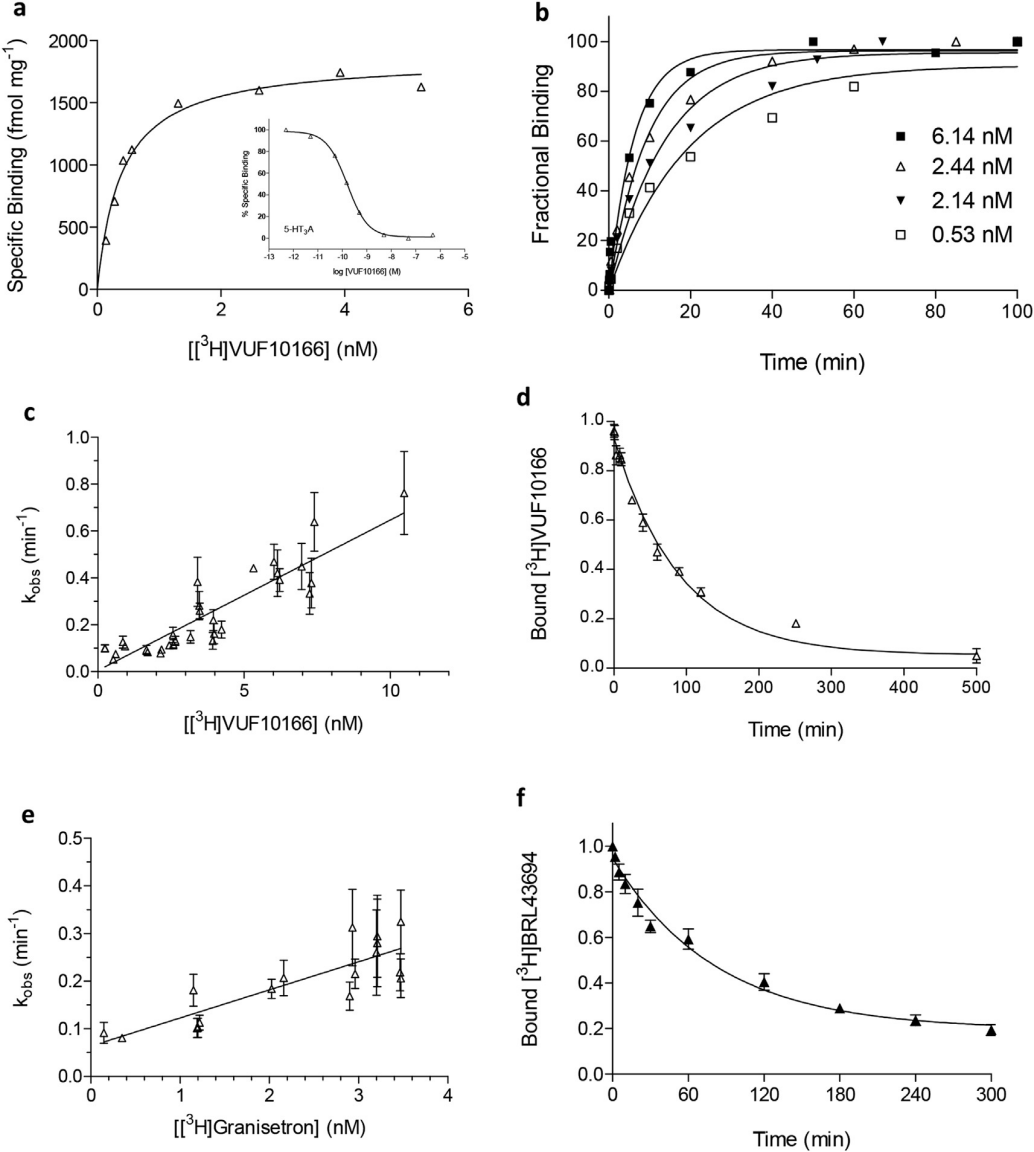
Radioligand binding at 5-HT_3_A receptors. (**a**) Representative binding curves for 5-HT_3_A receptors. *Inset* competition binding of unlabelled VUF10166 with [^3^H]granisetron. (**b**) Association of [^3^H]VUF10166 was fit with a mono-exponential function to yield *k*_obs_. (**c**) Linear regression was used to fit *k*_obs_ against the radioligand concentration, yielding the *k*_on_ (slope) and *k*_off_ (intercept at *y* = 0) values in Table 1. (**d**) Dissociation of [^3^H]VUF10166 was best fit with a single exponential (*k*_off_ = 0.011 ± 0.001 min^−1^, *n* = 4). (**e**) For [^3^H]granisetron, association was also best fit with mono-exponential functions that were used to plot *k*_obs_ against the concentration to yield the *k*_on_ and *k*_off_ values in Table 1. (**f**) Dissociation of [^3^H]granisetron (*k*_off_ = 0.011 ± 0.001 min^−1^, *n* = 5).

Mutagenesis was performed using the QuikChange method (Agilent Technologies Inc., California, USA) on human 5-HT3A cDNA (accession number: P46098) cloned into pcDNA3.1 (Invitrogen, Paisley, UK). Cysteine residues were substituted for amino acids throughout each of the binding loops A–E (Fig. 1). To facilitate comparisons with previous work, we use the numbering of the equivalent residues in the mouse 5-HT3A subunit; for human numbering 5 should be subtracted from each residue number.

### 2.3 Cell culture and transfection

Human embryonic kidney (HEK) 293 cells were maintained as monolayer cultures grown on 90 mm tissue culture plates in DMEM:F12 (Dulbecco's Modified Eagle Medium/Nutrient Mix F12 (1:1)) with GlutaMAX™ I media (Gibco BRL, Paisley, U.K.) containing 10% foetal calf serum (HyClone, Thermo Scientific, Cramlington, UK), at 37 °C and 7% CO_2_, with a humidified atmosphere. Cells were transfected using polyethyleneimine (PEI, Polysciences Inc., Eppelheim, Germany), and incubated for 2–3 days before harvesting.

### 2.4 Radioligand binding

Transfected HEK 293 cells were washed twice with phosphate buffered saline (PBS) at room temperature, scraped into 1 ml of ice-cold HEPES buffer (10 mM, pH 7.4), homogenised and frozen. After thawing, they were washed with HEPES buffer, resuspended, and 50 μg of cell suspension incubated in 0.5 ml HEPES buffer and the relevant concentration of radioligand at 0 °C. Non-specific binding was determined using 2 mM quipazine. Equilibrium reactions were incubated for at least 3 h for [^3^H] granisetron (63.5 Ci/mmol, PerkinElmer, Boston, Massachusetts, USA) and 48 h for [^3^H]VUF10166. Incubations were terminated by vacuum filtration onto GF/B filters pre-soaked in 0.3% polyethyleneimine, followed by three rapid washes with 3.5 ml ice cold buffer. Radioactivity was determined by scintillation in Ecoscint A (National Diagnostics, Atlanta, Georgia) using a Beckman LS6000SC (Fullerton, California, USA). Each method was performed on at least three independent cell samples on at least three separate days.

#### 2.4.1 Saturation binding

To construct saturation binding curves a range of [^3^H]granisetron (0.25–2 nM) or [^3^H]VUF10166 (0.04–50 nM) concentrations were used according to the conditions described above. Final counts were monitored to ensure that binding never exceeded 10% of the added concentrations of radioligands.

#### 2.4.2 Competition binding

Affinities of unlabelled Cys-loop receptor ligands were determined by adding a range (2 pM–2 mM) of concentrations to samples containing 0.2 nM [^3^H]VUF10166 or 0.7 nM [^3^H]granisetron for 5-HT_3_A receptors, and 0.6 nM [^3^H]VUF10166 or 0.7 nM [^3^H]granisetron for 5-HT_3_AB receptors.

#### 2.4.3 Kinetic measurements

To determine the association rate (*k*_on_), the observed association rate (*k*_obs_) was measured for a range of radioligand concentrations. The experiment was started (*t* = 0) by the addition of radioligand to 500 μl cell suspension in HEPES buffer and harvested at varying time points to construct association curves.

Dissociation was measured by allowing each radioligand to reach equilibrium according to the times described above and then adding a final concentration of 2 mM quipazine (∼*K*_d_ × 10^6^) to each tube for varying time periods.

### 2.5 Data analysis

All data were analysed using GraphPad Prism 4.03. Individual saturation binding experiments were fitted to Equ (1), and the values averaged to obtain mean ± sem:

(1) $$y=B_{\max}\times [L]\frac{K_{\text{d}}}{K_{\text{d}}+[L]}$$

where *B*_max_ is maximum binding at equilibrium, *K*_d_ is the equilibrium dissociation constant and [*L*] is the free concentration of radioligand. Individual competition binding experiments were analysed by iterative curve fitting using the following equation and the values averaged to obtain the mean ± sem:

(2) $$y=B_{\min}+\frac{B_{\max}-B_{\min}}{1+10^{\left[L\right]\cdot \log\;IC_{50}}}$$

where *B*_min_ is the non-specific binding, *B*_max_ is the maximum specific binding, [*L*] is the concentration of competing ligand and *IC*_50_ is the concentration of competing ligand that blocks half of the specific bound radioligand.

A simple bimolecular binding scheme for receptor and ligand can be represented as:

(3) $$L+R\overset{k_{\text{on}}}{\underset{k_{\text{off}}}{⇌}}LR$$

where *L* is the free ligand concentration, *R* receptor concentration, *LR* bound receptor concentration, and *k*_on_ and *k*_off_ microscopic association and dissociation rate constants. In a simple scheme such as this, the equilibrium dissociation constant (*K*_d_) is equal to the ratio of dissociation to association rate constants, such that:

(4) $$K_{\text{d}}=\frac{k_{\text{off}}}{k_{\text{on}}}$$

Dissociation data were fitted to either a single or double exponential decay to yield *k*_off_. Association data were fitted to a single exponential association to calculate *k*_obs_. If *k*_obs_ is plotted against the radioligand concentration, according to a simple model, the slope of this plot equals the association constant (*k*_on_) and the *y*-intercept of this line (at *x* = 0) is the dissociation constant (*k*_off_). *k*_on_ can also be calculated as described by Hill (Hill, 1909), where *k*_off_ is predetermined from radioligand dissociation rate experiments.

(5) $$k_{\text{on}}=\frac{k_{\text{obs}}-k_{\text{off}}}{\left[L\right]}$$

### 2.6 Homology modelling

The protein sequence of the human 5-HT3A subunit (accession number; P46098) was aligned with a tropisetron bound AChBP template (PDB ID; 2WNC) using FUGUE. Using Modeller 9.9, five homology models were generated using default parameters and the best model selected using Ramachandran plot analysis. For the ligand, the protonated form of VUF10166 was constructed in Chem3D Ultra 7.0 (CambridgeSoft, Cambridge, UK). The binding site was defined as being within 5 Å of the α-carbon of W183, a residue critical in the binding of other 5-HT_3_ competitive ligands. VUF10166 was docked into this site using the GOLD docking program (version 3.0, The Cambridge Crystallographic Data Centre, Cambridge, UK) with the GOLDScore function and default settings. Ten docking poses were generated for each of the five homology models and the poses visualised with PyMol v1.3.

## 3 Results

### 3.1 [^3^H]VUF10166 binding at 5-HT_3_A receptors

[^3^H]VUF10166 showed high affinity saturable binding at 5-HT_3_A receptors with low levels (<5%) of non-specific binding. The *K*_d_ value was similar to the *K*_i_ value from competition of unlabelled VUF10166 with [^3^H]granisetron (Fig. 1a, Table 1). *B*_max_ values for [^3^H]VUF10166 (2229 ± 158 fmol/mg, *n* = 6) were comparable to those with [^3^H]granisetron (2263 ± 101, *n* = 6) on paired samples, suggesting that both ligands bind to the same receptor population.

**Table 1:** Binding parameters for VUF10166 and BRL43694.

Receptor	*k*_on_ (M^−1^ min^−1^)	*k*_off_ (min^−1^)	*K*_d_(nM) (k_on_/k_off_)	*K*_d_ (nM) saturation	*K*_i_ (nM) competition^a^
*VUF10166*					
5-HT_3_A	6.25 × 10^7^	0.010	0.16	0.18 ± 0.04 (11)	0.24 ± 0.11 (12)
5-HT_3_AB	b6.15 × 10^7^ b7.23 × 10^6^	0.024 0.162	0.38 22.4	–	36.7 ± 12.4 (12)
*BRL43694*					
5-HT_3_A	5.90 × 10^7^	0.064	1.08	0.68 ± 0.05 (12)	–
5-HT_3_AB	1.20 × 10^8^	0.074	0.62	0.74 ± 0.10 (4)	–

a Competition binding was performed with [^3^H]BRL43694 and unlabelled VUF10166. *k*_on_ and *k*_off_ were calculated from plots of *k*_obs_ versus ligand concentration (Fig. 1, Fig. 5). – not determined.

b Not significantly different to 5-HT_3_A (*p* > 0.05, Student's *t*-test).

### 3.2 VUF10166 kinetic parameters at 5-HT_3_A receptors

Association curves for [^3^H]VUF10166 were best fit with a single exponential function (Fig. 1b), and the resultant rates (*k*_obs_) plotted against ligand concentration to yield *k*_on_ and *k*_off_ (Fig. 1c, Table 1). The value for *k*_on_ was similar to values determined directly from *k*_obs_ values using Equ (5) (8.24 × 10^7^ M min^−1^). Dissociation of [^3^H]VUF10166 in the presence of excess cold quipazine was also monophasic (Fig. 1d), with *k*_off_ values that were similar to those determined from plots of *k*_obs_ against ligand concentration (Table 1). *K*_d_ values calculated from these kinetic measurements (Equ (4)) were similar to those derived from the saturation and competition binding (Table 1). These results indicate [^3^H]VUF101666 binding can be best described by a simple bi-molecular binding scheme.

### 3.3 Specificity of binding

A range of competitive and non-competitive ligands of 5-HT_3_ and related Cys-loop receptors were tested for their ability to compete with [^3^H]VUF10166 binding (Table 2). All tested 5-HT_3_ receptor competitive ligands (agonists and antagonists) displaced specific [^3^H]VUF10166 binding. Binding was unaffected by the non-competitive ligands bilobalide, ginkgolide and picrotoxin, or the majority of competitive ligands of other Cys-loop receptors. Exceptions were strychnine (glycine receptor antagonist) and nicotine (nACh receptor agonist); these were later shown to also compete with [^3^H]granisetron.

**Table 2:** Competition of Cys-loop receptor ligands with [^3^H]VUF10166.

Compound	p*IC*_50_	
	5-HT_3_A	5-HT_3_AB
Allosetron	11.14 ± 0.01 (4)	11.15 ± 0.10 (4)
Quipazine	8.84 ± 0.03 (4)	8.60 ± 0.75 (5)
MDL72222	12.90 ± 0.01 (3)	13.23 ± 0.11(1)
*m*CPBG	7.49 ± 0.06 (4)	6.07 ± 0.20 (5)
Granisetron	10.48 ± 0.08 (4)	10.35 ± 0.10 (3)
*d*-Tubocurarine	5.41 ± 0.06 (4)	5.44 ± 0.30 (4)
5-HT	4.54 ± 0.07 (3)	4.49 ± 0.09 (3)
ACh	NB (4)	NB (3)
GABA	NB (4)	NB (3)
Glycine	NB (4)	NB (3)
Gabazine	NB (4)	NB (3)
Bicuculline	NB (5)	NB (3)
Strychnine	5.83 ± 0.09 (4)	6.26 ± 0.01 (2)
Picrotoxin	NB (3)	NB (3)
Bilobalide	NB (3)	NB (2)
Ginkgolide	NB (3)	NB (3)
Nicotine	6.81 ± 0.23 (4)	6.76 ± 0.09 (2)

Previously we showed that unlabelled VUF10166 does not compete with [^3^H]epibatidine at α7 nACh receptors (the closest pharmacologically related receptor) (Thompson et al., 2012). Here we performed saturation binding experiments on α7 nACh receptors using [^3^H]VUF10166 which revealed no specific saturable binding (data not shown).

These results show that classical 5-HT_3_ receptor competitive antagonists compete with [^3^H]VUF10166, showing it binds at the orthosteric site.

### 3.4 Granisetron binding at 5-HT_3_A receptors

To compare [^3^H]VUF10166 with a well-established 5-HT_3_ receptor competitive ligand, experiments were also conducted using [^3^H]granisetron. As expected, [^3^H]granisetron showed high affinity binding at 5-HT_3_A receptors (Table 1). Competition binding with a range of known 5-HT_3_ receptor agonists and antagonists gave *K*_i_ values similar to those determined using competition with [^3^H]VUF10166 (Table 3) and to those published elsewhere (Brady et al., 2001). Similar to [^3^H]VUF10166, nicotine and strychnine competed with [^3^H]granisetron.

**Table 3:** Competition of Cys-loop receptor ligands with [^3^H]BRL43694.

Compound	p*IC*_50_	
	5-HT_3_A	5-HT_3_AB
Quipazine	8.60 ± 0.02 (5)	8.12 ± 0.18 (5)
MDL72222	8.05 ± 0.09 (3)	7.96 ± 0.15 (3)
*m*CPBG	6.88 ± 0.13 (7)	6.64 ± 0.12 (5)
Granisetron	9.12 ± 0.05 (7)	9.14 ± 0.09 (4)
*d*-Tubocurarine	4.61 ± 0.17 (3)	4.29 ± 0.47 (3)
5-HT	6.38 ± 0.35 (6)	5.64 ± 0.45 (5)
Nicotine	6.01 ± 0.61 (3)	6.56 ± 0.12 (3)
Strychnine	4.30 ± 0.09 (3)	4.85 ± 0.19 (3)

[^3^H]granisetron association rates were best fit with a monophasic curve. *k*_obs_ increased with free ligand concentration and a straight line was fitted (Fig. 1e) to yield the *k*_on_ and *k*_off_ values in Table 1. *K*_d_ values calculated from these kinetic measurements (Equ (4)) were in agreement with affinities calculated from our saturation binding studies (Table 1). Dissociation was also monophasic and the rate agreed well with that from our *k*_obs_ versus concentration plots described above (Fig. 1f, Table 1).

These observations show that using a well-established radiolabelled 5-HT_3_ receptor antagonist ([^3^H]granisetron) we are able to accurately reproduce the binding characteristics reported elsewhere and, similar to [^3^H]VUF10166, they are consistent with a simple bi-molecular binding scheme.

### 3.5 Homology modelling & docking

**Fig. 2:**
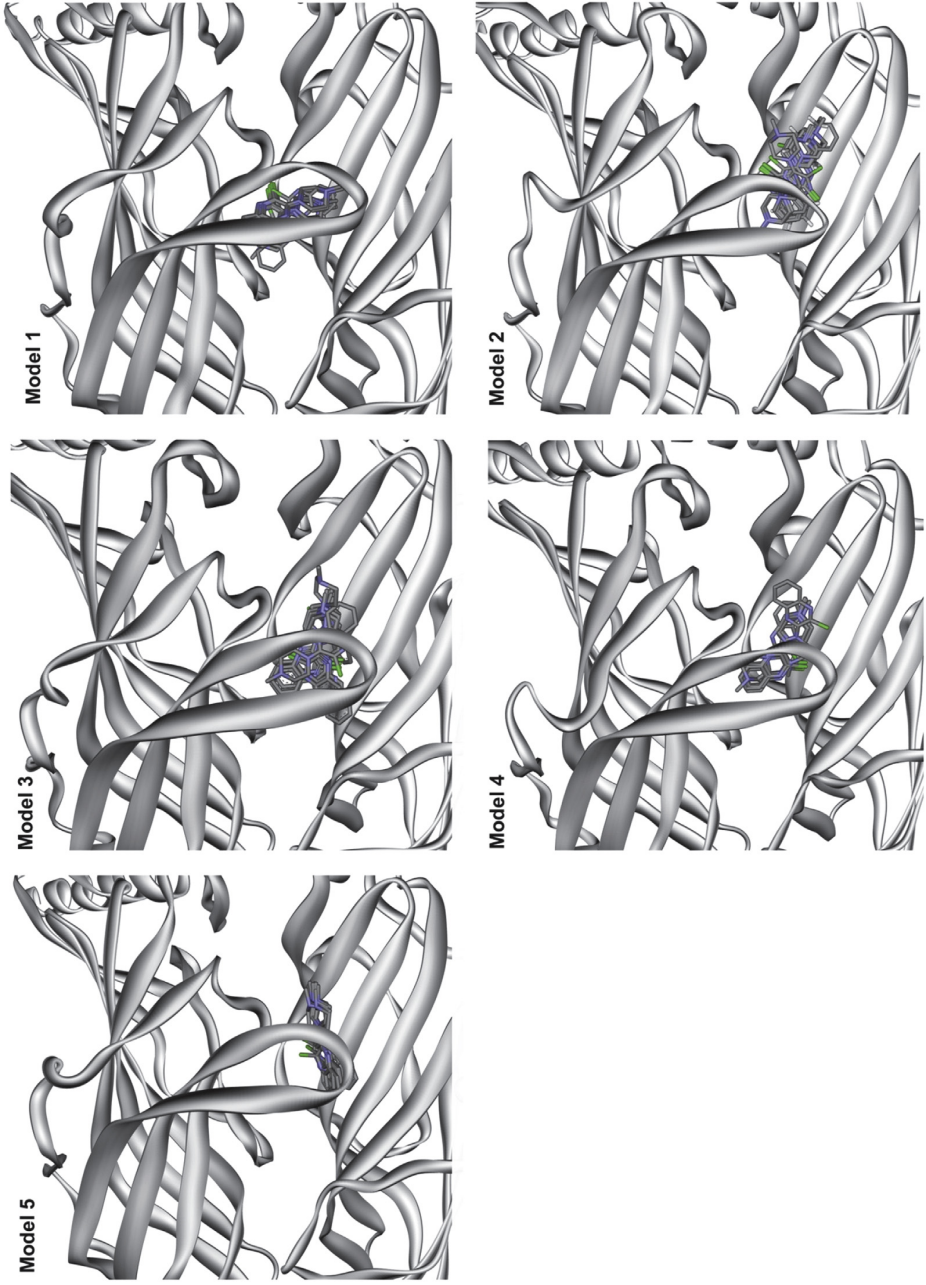
Predicted binding clusters for VUF10166 docked into five different homology models of the 5-HT_3_ receptor A+A− binding site. All 10 predicted ligand poses are shown for each model. The 5-HT_3_ receptor residues within 5 Å of VUF10166 in each of the docked poses are shown in Table 4.

**Fig. 3:**
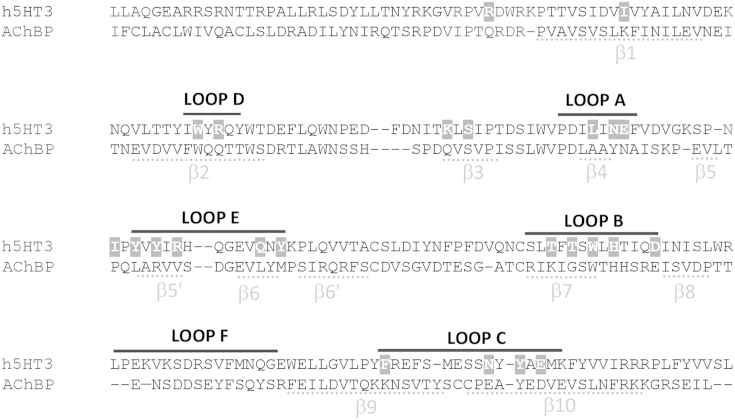
An amino acid sequence alignment showing the positions of residues mutated in this study (white text, grey boxes). The six recognised binding loops are indicated by black lines above the text. Positions of β-sheets are shown by grey lines beneath the text. Numbering of residues and structural features are taken from the AChBP protein crystal structure (Celie et al., 2004). The proteins are the human 5-HT3A subunit (P46098) and *Lymnaea stagnalis* AChBP (58154).

To gain insights into the residues that potentially interact with VUF10166 at the orthosteric site (A+A− interface), five 5-HT_3_A receptor homology models were generated and *in silico* docking of VUF10166 performed on each one (Fig. 2). A total of 50 docked poses were generated and for each of these the amino acids within 5 Å of VUF10166 were identified (Table 4). 26% of residues were common to all models, comparable to a previous docking study with granisetron, where 31% of residues were common to all of the predicted binding orientations (Thompson et al., 2005). A selection of these residues were chosen for mutagenesis based upon the following criteria, 1) side chains accessible to the ligand, 2) residues known to interact with other 5-HT_3_ ligands or, 3) residues present in a limited number of docked poses to provide support for specific orientations. Of the 39 amino acids identified, 23 were mutated to cysteine (Fig. 3); cysteine substitution of these residues was chosen as all of the Cys mutants have been previously shown to express on the cell-surface, and the residue positions have been similarly used for the study of our radioligand standard, [^3^H]granisetron (Thompson et al., 2005, Thompson et al., 2011).

**Table 4:**
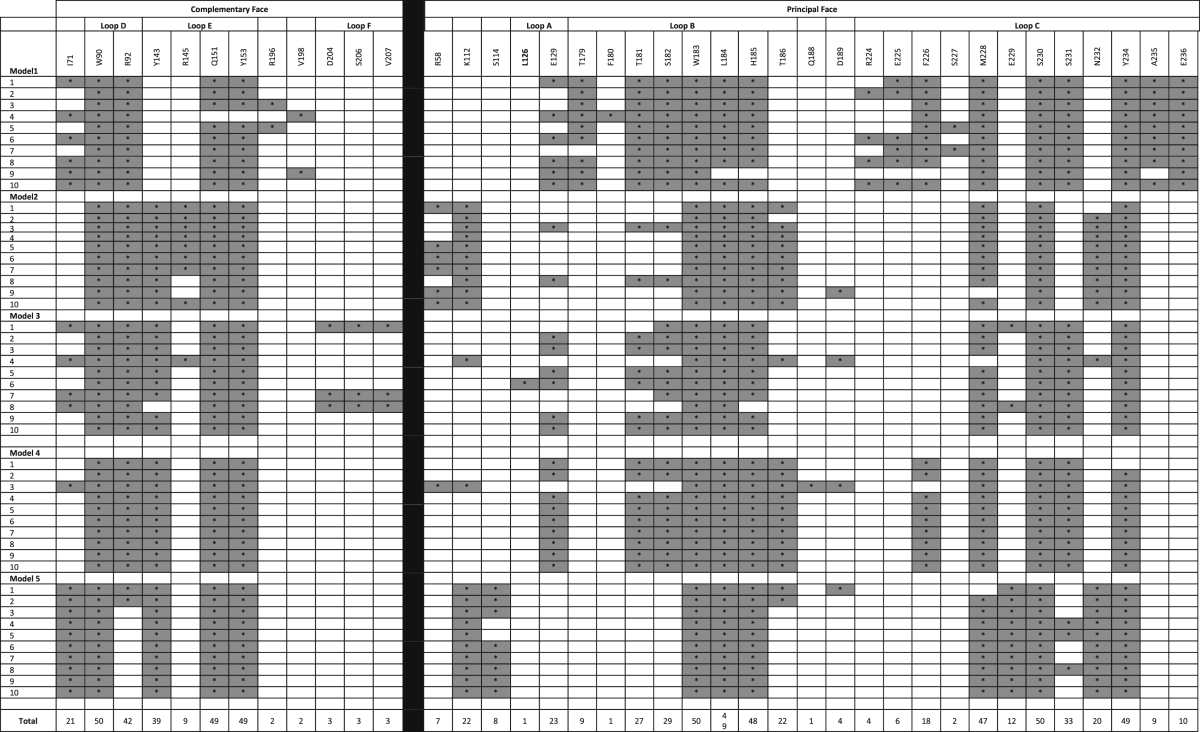
Residues within 5 Å of docked VUF10166 in 5 different homology models of the 5-HT_3_A receptor binding site.

### 3.6 Effects of mutations

**Fig. 4:**
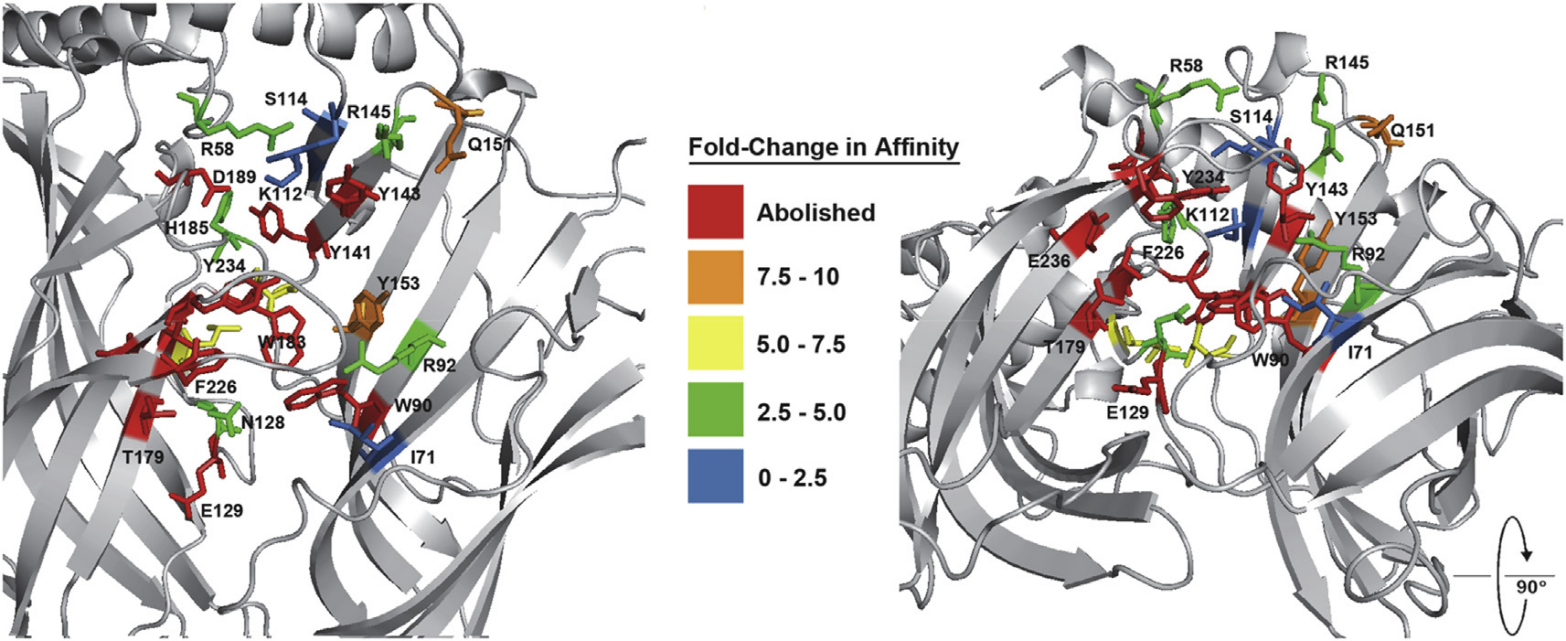
Binding site residues mutated in this study, colour coded according to the change in *K*_d_. A large number of residues that abolish binding (affinity > 10 nM) are clustered around loop B, with further significant changes in loop D (W90) and loop E (Y141 & Y143). Not all changes are likely to result from ligand interactions, such as the effect of D189C which is consistent with it maintaining the hydrogen bond network in the tight loop at the C-terminal end of loop B. (For interpretation of the references to colour in this figure legend, the reader is referred to the web version of this article.)

The binding affinity of [^3^H]VUF10166 at each of the mutant receptors is shown in Table 5, and their locations in Fig. 4. Changing 3 of the 23 residues resulted in no significant change in affinity, suggesting these residues do not play a role in ligand binding (I71, K112, S114). For the remaining 20 mutants there were differences in the binding affinities when compared to wild type receptors, indicating that these residues may have a role in VUF10166 binding. For 9 of these residues [^3^H]VUF10166 had reduced affinities (R92, L126, N128, I139, R145, Q151, Y153, H185, F226) and for 11 no saturable binding (*K*_d_ > 10 nM) was detected (R58, W90, E129, Y141, Y143, T179, T181, W183, H185, D189, Y234, E236). All these mutant receptors have been previously shown to express in oocytes (Thompson et al., 2012).

**Table 5:**
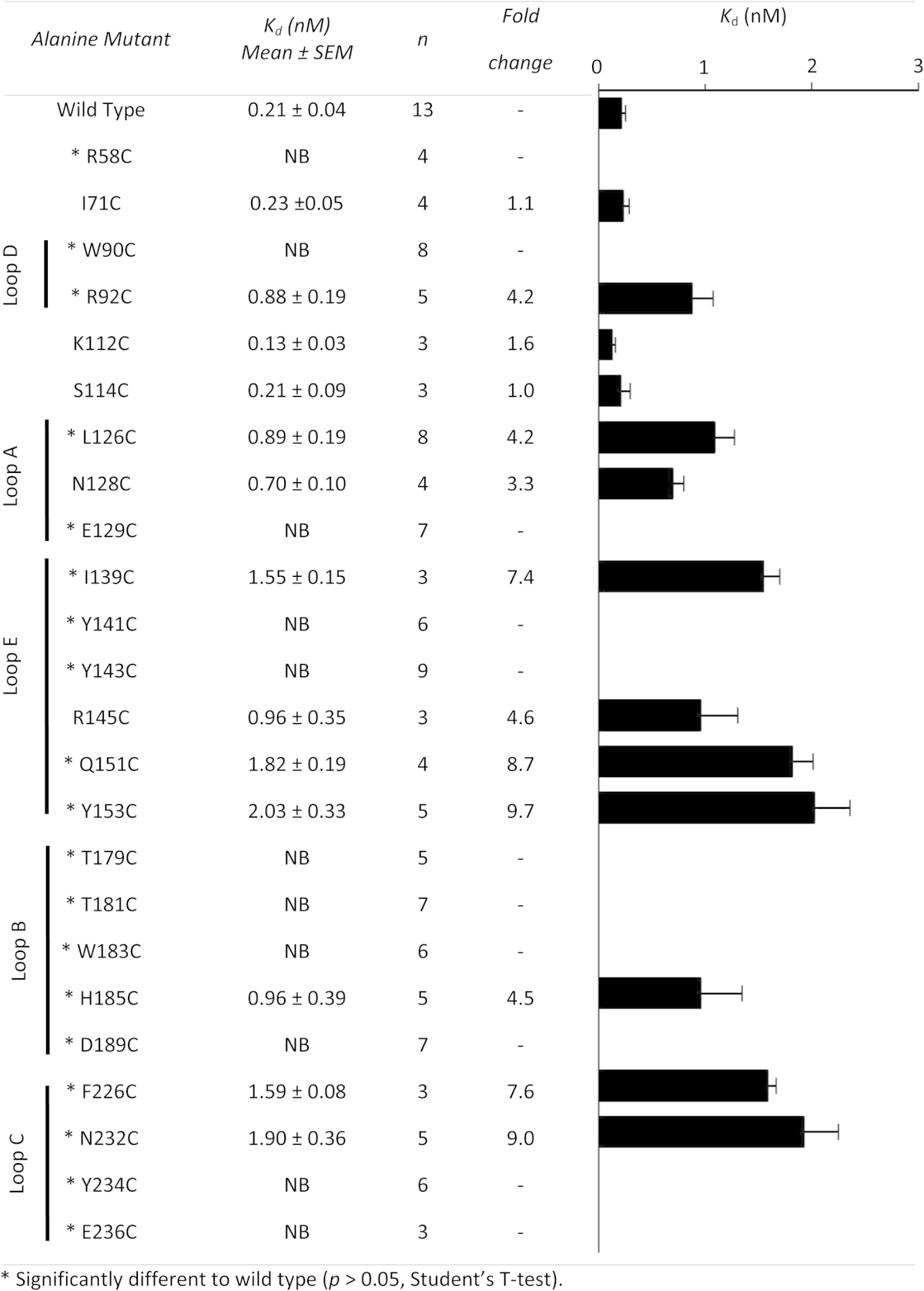
Saturation binding of [^3^H]VUF10166 at 5-HT_3_A receptor mutants.

These data show that [^3^H]VUF10166 binds to the orthosteric site and are consistent with our findings that [^3^H]VUF10166 competes with other 5-HT_3_ receptor competitive ligands.

### 3.7 VUF10166 binding at 5-HT_3_AB receptors

**Fig. 5:**
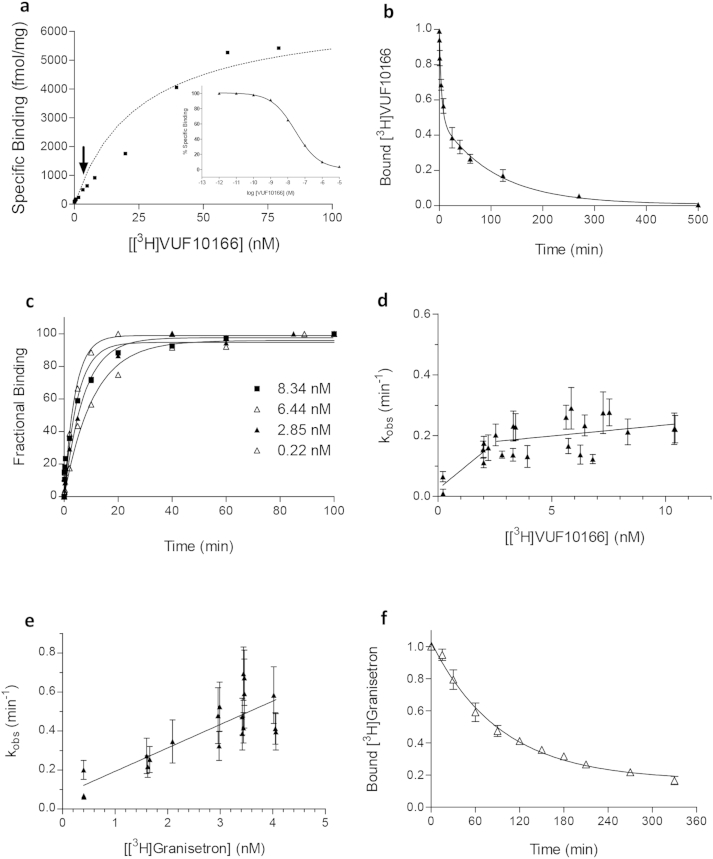
Radioligand binding at 5-HT_3_AB receptors. (**a**) Binding at 5-HT_3_AB receptors could not be well fit with a standard one site model; deviation occurs at a radioligand concentration of ∼3 nM (arrow). *Inset* competition binding of unlabelled VUF10166 with [^3^H]granisetron. (**b**) Dissociation was best fit with a double exponential at 5-HT_3_AB receptors (0.010 ± 0.003 min^−1^ and 0.227 ± 0.056 min^−1^, *n* = 8). (**c**) Association was mono-exponential, but a plot of *k*_obs_ against radioligand concentration. (**d**) revealed two components, showing that it was rate-limited at higher concentrations. (**e**) The association of [^3^H]granisetron was best fit with a mono-exponential function, but unlike [^3^H]VUF10166, the fit of *k*_obs_ against the radioligand concentration was linear at across all concentrations, yielding the values for *k*_on_ and *k*_off_ in Table 1. (**f**) Consistent with this plot, dissociation of [^3^H]granisetron was also best described by a single exponential function (*k*_off_ = (0.012 ± 0.002 min^−1^, *n* = 5)) that was not significantly different to 5-HT_3_A receptors (*p* > 0.05, Student's *t*-test).

VUF10166 was previously shown to discriminate between 5-HT_3_ receptors subtypes (Thompson et al., 2012) and so binding properties of the new radioligand were also tested at 5-HT_3_AB receptors. [^3^H]VUF10166 showed high affinity binding at 5-HT_3_AB receptors, but unlike at 5-HT_3_A receptors, it was complex and could not be fit with a single site model (Fig. 5a). Dissociation of [^3^H]VUF10166 at these receptors was best fit with a double exponential curve, which contained both a fast and a slow component; the latter was not significantly different (*p* < 0.05) to the single rate measured at 5-HT_3_A receptors (Fig. 5b, Table 1). Association curves were monophasic (Fig. 5c), but when *k*_obs_ was plotted against radioligand concentration, the data were also best approximated by a two site fit (Fig. 5d, Table 1). At concentrations of [^3^H]VUF10166 < 3 nM the *k*_off_ and *k*_on_ values were similar to 5-HT_3_A receptors; below 3 nM, average *k*_on_ values determined from *k*_obs_ (Equ (5)) were also similar to 5-HT_3_A receptors (8.77 × 10^7^ M min^−1^). At concentrations >3 nM, *k*_off_ and *k*_on_ had slower rates that yielded a *K*_d_ (22.4 nM; Equ (4)) close to the value from competition binding (36.7 nM; Table 1). Competition binding with a range of ligands was performed using 0.6 nM [^3^H]VUF10166 and *K*_i_ values were similar to values at 5-HT_3_A receptors (Table 2).

These results show that [^3^H]VUF10166 has different binding properties at 5-HT_3_A and 5-HT_3_AB receptors. In the latter effects are complex and some only become apparent at higher concentrations of [^3^H]VUF10166.

### 3.8 Granisetron binding at 5-HT_3_AB receptors

Unlike [^3^H]VUF10166, [^3^H]granisetron saturation binding at 5-HT_3_AB receptors yielded *K*_d_ values that were the same as those at 5-HT_3_A receptors, as reported elsewhere (Table 1) (Brady et al., 2001). Association (Fig. 5e), dissociation (Fig. 5f) and *K*_i_ values from competition binding (Table 3) were also the same as those at 5-HT_3_A receptors.

These results show that the binding properties of [^3^H]granisetron are the same at 5-HT_3_A and 5-HT_3_AB receptors unlike those of [^3^H]VUF10166.

## 4 Discussion

[^3^H]VUF10166 binds specifically and with high affinity to 5-HT_3_A and 5-HT_3_AB receptors, with evidence of a second, lower affinity, binding site in 5-HT_3_AB receptors. The effects of this second site are apparent at concentrations of [^3^H]VUF10166 > 3 nM, and are consistent with previous work that identified an additional allosteric binding site for unlabelled VUF10166 at the A+B− interface (Thompson et al., 2012). Docking of this competitive ligand into the orthosteric (A+A−) binding site, combined with data from mutagenesis, suggest that VUF10166 is oriented with its quinoxaline rings close to W183 and its basic nitrogen extended towards loop E. Individual residues, many of which have been previously shown to be important in studies of other 5-HT_3_ receptor ligands (including *d*-tubocurarine, granisetron, lerisetron, *meta*-chlorophenylbiguanide and tropisetron) are also important for VUF10166 binding (Hope et al., 1999, Mochizuki et al., 1999, Venkataraman et al., 2002a, Price and Lummis, 2004). The residues are discussed in more detail below.

### 4.1 The role of loop A residues

VUF10166 binding was abolished by Cys substitution of E129, slightly modified by L126C (∼4 fold change in *K*_d_) and not altered by N128C. E129 was previously identified as an important 5-HT_3_ receptor binding residue and may form a hydrogen bond with bound ligand, which is consistent with our data (Price et al., 2008). However, data from 5HTBP (a modified AChBP with high affinity binding for 5-HT_3_ receptor ligands) suggest that E129 may hydrogen bond with the side chain of T179 (Kesters et al., 2013), and therefore might have primarily a structural role. L126 may also have a structural role but is less important as the effects of altering this residue were small, while N128 has been shown to play a role in gating but not binding (Price et al., 2008, Kesters et al., 2013).

### 4.2 The role of loop B residues

Loop B has been previously identified as both a critical structural component of the binding pocket, and it contributes to ligand binding. W183 is especially important as a constituent of the ‘aromatic box’ that exists in all Cys-loop receptor binding sites (Beene et al., 2002, Thompson et al., 2008b, Duffy et al., 2012). Other residues (T179, H185, D189) are known to stabilise the binding site structure via hydrogen bonds (Thompson et al., 2008b, Kesters et al., 2013). It is therefore not surprising that all our loop B mutations altered or abolished [^3^H]VUF10166 binding and we suggest that T181 and W183 interact with VUF10166 while T179, H185 and D189 have a structural role.

### 4.3 The role of loop C residues

F226 and Y234 are also constituents of the aromatic box and mutations here alter or eliminate VUF10166 binding. F226A has no effect on granisetron binding affinity, indicating this residue is more important for VUF10166 binding (Thompson et al., 2005). In 5HTBP Y234 (Y193) interacts with 5-HT and also contributes to a conserved water network that stabilises the granisetron-bound structure (Kesters et al., 2013); a conserved water network is also seen at this location in many AChBP crystal structures and may be important in many Cys-loop receptors. E236C also abolished VUF10166 binding, consistent with studies where substitutions affect binding of both GR65630 and granisetron, as well as altering the maximal current and *EC*_50_ of 5-HT responses (Schreiter et al., 2003, Nyce et al., 2010). However E236 mutations may adversely affect the correct assembly of the binding site rather than interfering with specific ligand interactions as Nyce et al. (2010) and Schreiter et al. (2003) showed that some E236 mutant receptors are trapped within the cell. As this hypothesis is supported by the lack of interactions in the 5HTBP structure, we consider it unlikely that E236 contributes to VUF10166 binding (Kesters et al., 2013).

### 4.4 The role of loop D residues

W90 is another aromatic box residue that contributes to binding. In 5HTBP the equivalent residue (W53) is involved in van der Waals interactions with granisetron and W90 may have a similar role in binding VUF10166 (Spier and Lummis, 2000, Price and Lummis, 2004, Thompson et al., 2005, Yan and White, 2005). Substitutions at W90 decrease the affinity of other potent 5-HT_3_ receptor-specific ligands such as curare, lerisetron and 5-HT (Yan et al., 1999, Venkataraman et al., 2002a) R92 interacts with granisetron in 5HTBP (R55), and the effects of its substitution on the affinity of VUF10166, ondansetron, granisetron and MDL72222, suggest an interaction with all of these ligands (Thompson et al., 2005, Yan and White, 2005).

### 4.5 The role of loop E residues

All of the mutations in loop E (Y141, Y143, R145, Q151, Y153) caused significant changes to [^3^H]VUF10166 binding. In the 5HTBP crystal structure granisetron does not extend towards loop E, but instead lies horizontally between loops B and D, similar to the orientations of the closely related ligands tropisetron (2WNC) and cocaine (2PGZ) in AChBP. In contrast, in 5HTBP 5-HT hydrogen bonds with the backbone carbonyls of I104 (Y141 in 5-HT_3_) and I116 (Y153), and has hydrophobic interactions with M114 (Q151), explaining why 5-HT activation is strongly affected by mutations at these locations, but effects on granisetron are less apparent (Venkataraman et al., 2002b, Price and Lummis, 2004, Thompson et al., 2011, Kesters et al., 2013). Here the affinity of VUF10166 was decreased 10-fold by Y153C and abolished by Y143C, indicating that bound VUF10166 extends towards, and may interact with, loop E residues. As VUF10166 is also a low efficacy partial agonist at μM concentrations, and must therefore induce the same structural changes as 5-HT, it is likely it adopts an orientation that at least partially mimics that of 5-HT.

### 4.6 The orientation of VUF10166 in the ligand binding pocket

Our results show that VUF10166 binding is affected by many of the residues previously identified as important for binding 5-HT_3_ receptor antagonists, while mutation of R58, I71, K112 and S114, which are close to VUF10166 in models 1, 2 and 5, did not alter its affinity, suggesting that these models are less probable. Also in model 1 the predicted ligand orientations do not extend towards Loop E and yet residues here were important for VUF10166 binding. Similarly R145 is within 5 Å of VUF10166 in model 2, but our mutagenesis data show that altering this residue has little effect on binding affinity. Model 3 seems unlikely as these poses are positioned closer to the complimentary face of the binding site, and do not significantly interact with key principal face residues such as T181, W183 and Y234. Models 4 and 5 have quite similar docked poses with only F226 distinguishing them; F226C mutant receptors had a 7-fold lower affinity than wild type receptors suggesting that this residue is close enough to interfere with VUF10166 binding, which would best fit with model 4.

**Fig. 6:**
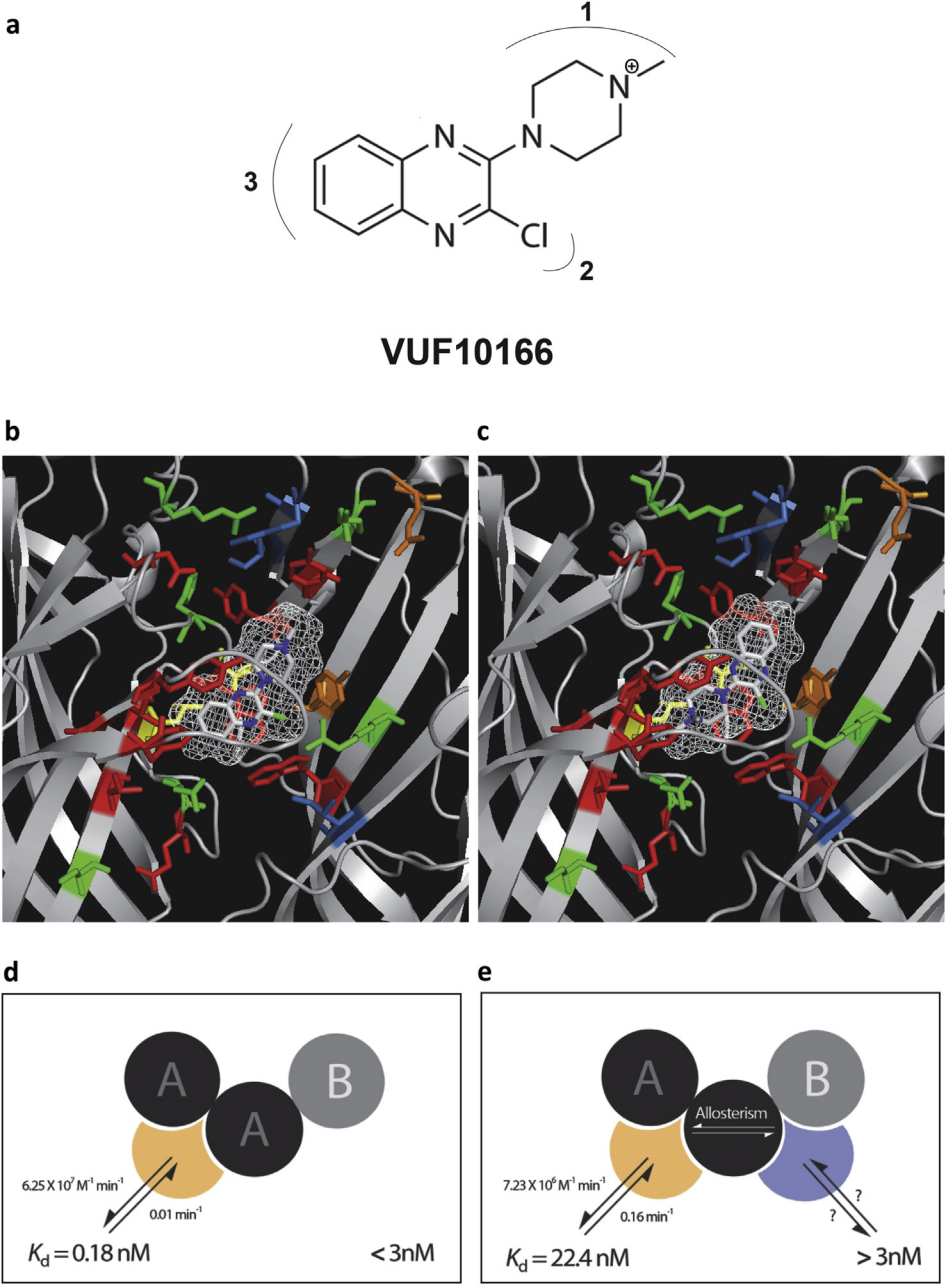
Chemical structure of VUF10166 and its binding mode. (**a**) Three regions of the ligand are identified and are described in the text. Its protonation site, which is also its tritiation site, is indicated. (**b**–**c**) The volume occupied by the two main docked pose clusters in model 4. In (c) cation–π interactions are possible with W183 (5.06 Å away) and Y234 (4.46 Å). VUF10166 is shown as a stick and wire mesh representation (white), with the residues mutated in this study colour coded similar to Fig. 4. (**d**–**e**) Cartoons showing our interpretation of the binding to heteromeric receptors. Below 3 nM, VUF10166 binds to a single population of binding sites at the A+A− interface of both 5-HT_3_A and 5-HT_3_AB receptors; consequently, at these concentrations both receptors share common values for *k*_on_ and *k*_off_. At concentrations of VUF10166 > 3 nM, binding also occurs at a second A+B− binding site and allosterically influences the adjacent A+A− site; therefore, additional rates are apparent and saturation binding is confounded by rates associated with multiple binding sites and allosteric interactions. (For interpretation of the references to colour in this figure legend, the reader is referred to the web version of this article.)

In previous work we presented a structure-activity study (SAR) of VUF10166 analogues (Verheij et al., 2012, Thompson et al., 2013) and the active analogues from these studies would fit well into model 4 in two distinct orientations (Fig. 6). These data showed substitutions of the chlorine atom in VUF10166 (Fig. 6a, region 2) are poorly tolerated, suggesting an important interaction at this location; in both poses in Fig. 6 the chlorine atom is closely located to R92 and W90. In contrast, substitutions in regions 1 and 3 are fairly well tolerated, providing that they are not too large; neither of the poses in Fig. 6 are sterically restricted around these regions of VUF10166. The poses also explain the importance of the charged *N*-methylpiperazine nitrogen atom, as there are possible cation–π interactions with W183 and Y234 in one pose, with these residues contributing to π–π stacking of the quinoxaline ring in the other.

We therefore suggest that the docked poses in model 4 are most consistent with the mutagenesis data described here and our previously published SAR. It is difficult to predict whether the *N*-methylpiperazine ring or the quinoxaline ring is positioned toward loop E, but the orientation in Fig. 6c is most reminiscent of varenicline co-crystallised into AChBP (PDBID = 4AFG & 4AFT) and 5-HT in 5HTBP (2YMD), both of which are agonists at 5-HT_3_ receptors (Billen et al., 2012, Rucktooa et al., 2012). This similarity in orientation may explain why VUF10166 also displays partial agonist activity (Thompson et al., 2012). However, it should be stressed that we must exercise caution when making these predictions as the physiological relevance of these structures have not yet been fully ascertained, for example three ligand molecules have been observed in a single AChBP binding site, something we would not have predicted (Brams et al., 2011, Stornaiuolo et al., 2013).

## 5 Conclusion

Our results show that VUF10166 interacts with several of the core binding site residues found at the A+A− interface and, combined with homology modelling and ligand docking, we propose it adopts an orientation similar to that of other 5-HT_3_ receptor agonists in AChBP and 5HTBP crystal structures. At 5-HT_3_ receptors our kinetic measurements are consistent with a single A+A− binding site, but at 5-HT_3_AB an additional fast component is seen. This is consistent with the lower affinity of VUF10166 for the 5-HT_3_AB receptor and is likely to result from an allosteric effect that is evident when the concentration of VUF10166 exceeds 3 nM (as summarised in Fig. 6).
